# The Influence of Prenatal Exposure to Quetiapine Fumarate on the Development of Dopaminergic Neurons in the Ventral Midbrain of Mouse Embryos

**DOI:** 10.3390/ijms232012352

**Published:** 2022-10-15

**Authors:** Walaa F. Alsanie, Sherin Abdelrahman, Majid Alhomrani, Ahmed Gaber, Ebtisam Abdulah Alosimi, Hamza Habeeballah, Heba A. Alkhatabi, Raed I. Felimban, Charlotte A. E. Hauser, Hossam H. Tayeb, Abdulhakeem S. Alamri, Abdulwahab Alamri, Bassem M. Raafat, Khaled A. Alswat, Yusuf S. Althobaiti, Yousif A. Asiri

**Affiliations:** 1 Department of Clinical Laboratories Sciences, The Faculty of Applied Medical Sciences, Taif University, P.O. Box 11099, Taif 21944, Saudi Arabia; 2 Centre of Biomedical Sciences Research (CBSR), Deanship of Scientific Research, Taif University, P.O. Box 11099, Taif 21944, Saudi Arabia; 3 Laboratory for Nanomedicine, Division of Biological and Environmental Science and Engineering (BESE), King Abdullah University of Science and Technology (KAUST), Thuwal, Jeddah 23955, Saudi Arabia; 4 Department of Biology, College of Science, Taif University, P.O. Box 11099, Taif 21944, Saudi Arabia; 5 Department of Medical Laboratory Technology, Faculty of Applied Medical Sciences in Rabigh, King Abdulaziz University, Jeddah 21589, Saudi Arabia; 6 Department of Medical Laboratory Sciences, Faculty of Applied Medical Sciences, King Abdulaziz University, Jeddah 21589, Saudi Arabia; 7 Center of Excellence in Genomic Medicine Research (CEGMR), King Abdulaziz University, Jeddah 21589, Saudi Arabia; 8 King Fahd Medical Research Centre, Hematology Research Unit, King Abdulaziz University, Jeddah 21589, Saudi Arabia; 9 Center of Innovation in Personalized Medicine (CIPM), 3D Bioprinting Unit, King Abdulaziz University, Jeddah 21589, Saudi Arabia; 10 Computational Bioscience Research Center (CBRC), King Abdullah University of Science and Technology (KAUST), Thuwal, Jeddah 23955, Saudi Arabia; 11 Nanomedicine Unit, Center of Innovation in Personalized Medicine (CIPM), King Abdulaziz University, Jeddah 21589, Saudi Arabia; 12 Department of Pharmacology and Toxicology, College of Pharmacy, University of Hail, Hail 55211, Saudi Arabia; 13 Department of Radiological Sciences, College of Applied Medical Sciences, Taif University, P.O. Box 11099, Taif 21944, Saudi Arabia; 14 Department of Internal Medicine, School of Medicine, Taif University, P.O. Box 11099, Taif 21944, Saudi Arabia; 15 Department of Pharmacology and Toxicology, College of Pharmacy, Taif University, P.O. Box 11099, Taif 21944, Saudi Arabia; 16 Addiction and Neuroscience Research Unit, Taif University, P.O. Box 11099, Taif 21944, Saudi Arabia; 17 Department of Clinical Pharmacy, College of Pharmacy, Taif University, P.O. Box 11099, Taif 21944, Saudi Arabia

**Keywords:** quetiapine fumarate, dopaminergic neurons, fetal neurodevelopment, ventral midbrain, embryonic neurons

## Abstract

The effects of second-generation antipsychotics on prenatal neurodevelopment, apoptotic neurodegeneration, and postnatal developmental delays have been poorly investigated. Even at standard doses, the use of quetiapine fumarate (QEPF) in pregnant women might be detrimental to fetal development. We used primary mouse embryonic neurons to evaluate the disruption of morphogenesis and differentiation of ventral midbrain (VM) neurons after exposure to QEPF. The dopaminergic VM neurons were deliberately targeted due to their roles in cognition, motor activity, and behavior. The results revealed that exposure to QEPF during early brain development decreased the effects of the dopaminergic lineage-related genes *Tyrosine hydroxylase* *(Th)*, *Dopamine receptor D1* (*Drd1*), *Dopamine transporter* (*Dat*), *LIM homeobox transcription factor 1 alfa* (*Lmx1a)*, and *Cell adhesion molecule L1* (*Chl1)*, and the senescent dopaminergic gene *Pituitary homeobox 3* (*Pitx3)*. In contrast, *Brain derived neurotrophic factor* (*Bdnf)* and *Nuclear receptor-related 1 (Nurr1)* expressions were significantly upregulated. Interestingly, QEPF had variable effects on the development of non-dopaminergic neurons in VM. An optimal dose of QEPF (10 µM) was found to insignificantly affect the viability of neurons isolated from the VM. It also instigated a non-significant reduction in adenosine triphosphate formation in these neuronal populations. Exposure to QEPF during the early stages of brain development could also hinder the formation of VM and their structural phenotypes. These findings could aid therapeutic decision-making when prescribing 2nd generation antipsychotics in pregnant populations.

## 1. Introduction

In women of reproductive age, atypical antipsychotics are routinely prescribed to manage bipolar disorder (BD). Between the early 2000s and 2007, prenatal atypical antipsychotic drug use doubled in the United States [1]. Various therapeutic choices exist globally for managing and treating a spectrum of psychotic disorders, including both first- and second-generation antipsychotic drugs [2,3]. The use of antipsychotic drugs during pregnancy remains controversial, mainly due to insufficient data on the exposure and outcomes to make accurate assessments of the risks [4]. There are limited treatment guidelines for pregnant women with newly diagnosed schizophrenia and those accidentally exposed to antipsychotic drugs during early pregnancy [5].

First-generation antipsychotics, such as haloperidol, are known to cause several side effects, such as the “EPS phenomenon” (extrapyramidal symptoms), gait disorders, and reproductive offset, including fetal toxicity, particularly when being used for the long term [6]. Acting primarily on the cell architecture within the central nervous system (CNS), antipsychotic drugs can easily cross the placental exchange barrier and blood–brain barrier as they are hydrophobic drugs with an affinity for brain cells. Consequently, these drugs can negatively impact the developing fetus. A few reports have described the effects of haloperidol on the developing fetal brain, such as altering morphology and changing cell architecture, including causing the loss of functional neuron volume, thereby causing functional alterations in the brain [7,8]. Drug therapy selection, continuation, or discontinuation in pregnant individuals is challenging for healthcare professionals. There is insufficient evidence to support the empirical use of these drugs, and the risk of developmental neurotoxicity has not been weighed against the benefits of therapy. To overcome this imbalance between the benefits of therapy and risks of teratogenic adverse effects, second-generation atypical antipsychotics like risperidone, olanzapine, and quetiapine were approved and soon became the first-line treatment for pregnant women with psychotic illnesses. Although evidence has repeatedly reinforced the superior safety of these newer drugs over their first-generation counterparts, there is no literature to support their use in pregnant populations, considering the risk of fetal toxicity [9].

In a study conducted in the United States (2006–2011), quetiapine fumarate (QEPF) was the most frequently prescribed second-generation antipsychotic during pregnancy [10]. Subsequently, it was found that in women of reproductive age in Denmark (2009–2011), the use of QEPF increased by 83%. A study on pregnant women in Australia (2000–2011) showed a similar trend, with QEPF use increasing thrice [5,11]. Although the Food and Drug Administration (FDA) has approved QEFP for BD and schizophrenia only, it has been widely used in other psychotic disorders, such as insomnia, unipolar depression, and generalized anxiety disorder (GAD) [12]. Pregnant women using atypical antipsychotics could be at risk of fetal abnormalities, although sufficient pharmacological data to support this hypothesis is lacking [13].

Cognition and motor activity are two important brain functions regulated by coordinating dopaminergic neurons in the ventral midbrain (VM). Given the lack of documented evidence on the effects of QEPF on the fetal brain, particularly on dopaminergic VM neurons, this study aimed to inspect whether QEPF affects developmental cues in the VM neurons of embryonic mice. VM neurons have a known role in learning, motivation, reward stimulation, coordination, and movement control, as well as having key roles in motor function and cognition. Disruption of the developmental cues of VM neurons could alter their functions and roles in adult brains. To closely mimic the native brain tissue, we employed a 3D cell culture technique using a tetrameric self-assembling peptide-based scaffold. These peptides create nanofibrous networks that resemble the extracellular matrix (ECM) structure of collagen [14,15]. In previous studies, Tetrameric self-assembling peptides have proven to be suitable scaffolds for developing efficient 3D neuronal models [15,16]. Neurotransmitters and neuromodulators, such as dopamine, are involved in a wide range of cognitive and behavioral functions in the adult brain, such as movement, thinking, and feeling pleasure. Dopamine-based signaling is important for forebrain development and circuit setup [17]. Previous literature has reported the use of quetiapine to raise the levels of noradrenaline and dopamine in the prefrontal cortex and caudate nucleus [18,19]. To our knowledge, this is the first study to investigate the mechanisms by which QEPF exposure affects the growth of isolated VM neurons in mouse embryos.

## 2. Results

### 2.1. The Effects of QEPF on the Metabolic Activity of VM Neurons

**Figure 1:**
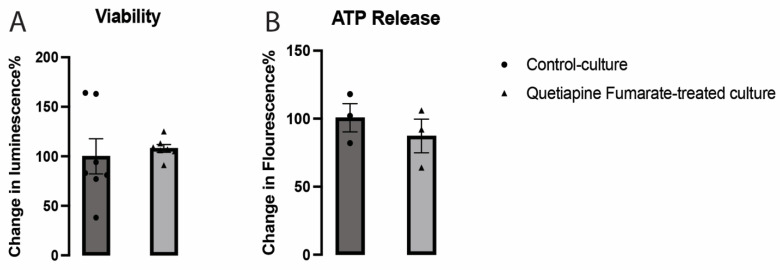
Quetiapine Fumarate (QEPF) did not significantly affect the viability (**A**) and ATP release (**B**) of VM neurons. Data expressed as mean ± SEM, *n* = 3 technical replicates, 7 biological replicates (Viability), and 3 biological replicates (ATP release).

Neuronal viability in cells treated with QEPF was unchanged compared to that in control cells (Figure 1A), thereby proving that the therapeutic dosage of 10 µM was not harmful to neuronal cells. Furthermore, the metabolic mechanisms of VM neurons were not significantly affected by QEPF (Figure 1B).

### 2.2. The Effects of QEPF on the Morphogenesis of Dopaminergic VM Neurons

**Figure 2:**
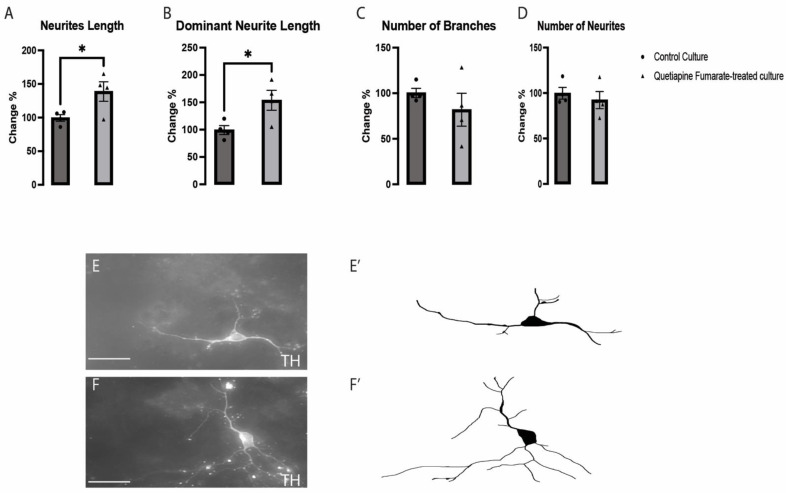
Quetiapine fumarate (QEPF) effects on length of neurites (**A**), dominant neurite length (**B**), number of branches (**C**), and number of neurites (**D**). Representative images and illustrations for dopaminergic VM neurons immunolabeled with TH in both groups: control (**E**,**E’**) and QEPF-treated (**F**,**F’**) show the increase in neurites elongation in response to QEPF exposure. Data are represented as the mean ± SEM (*n* = 3 technical replicates, 4 biological replicates). * *p* < 0.05.

The impact of QEPF on the development of dopaminergic VM neurons, which are positive for tyrosine hydroxylase (TH), was evaluated in labeled cultures. Although total and dominant neurite lengths (Figure 2A,B) were shown to have undergone structural modifications in cells treated with QEFP, there were no discernible variations in the numbers of branches or neurites in QEPF-treated cultures when compared to those in control cultures (Figure 2C,D). These findings suggest that exposure to QEPF did affect neuronal differentiation and morphogenesis, which were subsequently investigated by analyzing gene expression affecting differentiation signals.

### 2.3. QEPF Does Not Affect the Morphogenesis of Non-Dopaminergic VM Neurons

**Figure 3:**
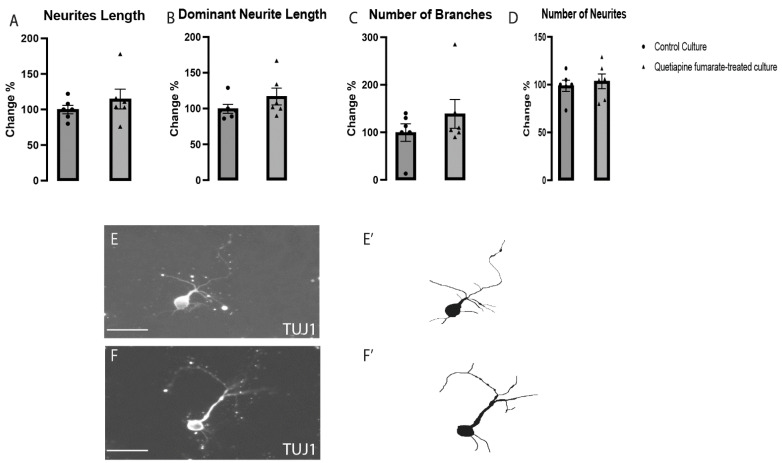
QEPF exposure did not affect the morphology of non-dopaminergic VM neurons (**A**–**D**). Representative images and illustrations for non-dopaminergic VM neurons immunolabeled with TUJ1 in both groups: control (**E**,**E’**) and QEPF-treated (**F**,**F’**) show no significant differences between groups. Data expressed as mean ± SEM, *n* = 3 technical replicates, 6 biological replicates.

To ascertain whether QEPF had a general influence on all VM neurons or whether it was restricted to dopaminergic VM neurons, the effects of QEPF on the morphogenesis of non-dopaminergic VM neurons (TH−/TUJ1+) were assessed in labeled cultures. No discernible changes were observed between the control and QEPF-treated cultures in terms of neurite morphology, total neurite length, dominant neurite length, number of branches, or neurite count (Figure 3). According to the aforementioned findings, QEPF does not substantially affect the differentiation and morphogenesis of non-dopaminergic neurons in the VM.

### 2.4. QEPF Induces Changes in the Expression of Crucial Dopaminergic-Related Genes in VM Neurons

**Figure 4:**
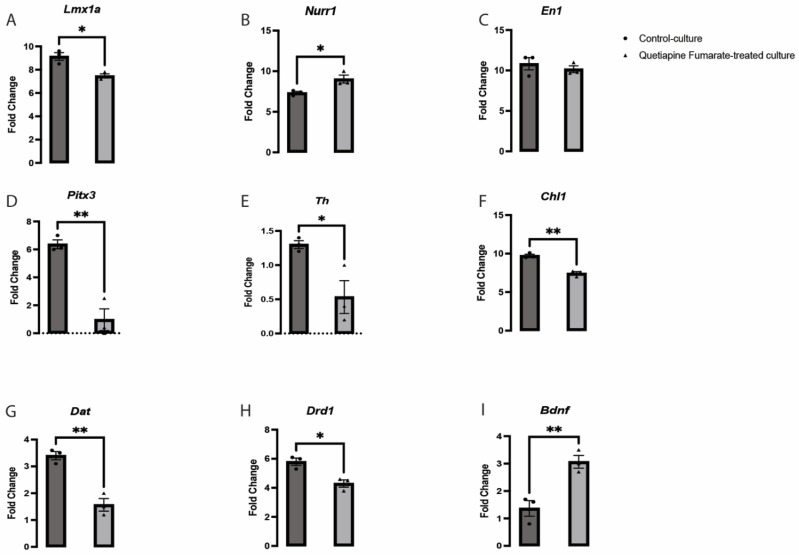
QEPF exposure caused a significant change in the expression of various dopaminergic VM-related genes. The expressions of *Lmx1a* (**A**), *Nurr1* (**B**), *Pitx3* (**D**), *Th* (**E**), *Chl1* (**F**), *Dat* (**G**), *Drd1* (**H**), and *Bdnf* (**I**) were altered by the exposure to QEPF. However, the expression of *En1* (**C**) *was* not affected by QEPF exposure. Data are represented as mean ± SEM, *n* = 3 technical replicates, 3 biological replicates. * *p* < 0.05, ** *p* < 0.01.

Several early dopaminergic fate-determining genes, including *Wnt family member 5A (Wnt5a), Pituitary homeobox 3* (*Pitx3),* and *Tyrosine hydroxylase* *(Th)*, have been identified as key regulators of neurogenesis. However, these genes do not govern all the factors involved in dopaminergic VM development [20,21,22]. The gene *LIM homeobox transcription factor 1 alfa/beta* (*Lmx1a/b)* is vital for VM neuronal generation [23,24]. A significant decrease was observed in *Lmx1a* expression in the QEPF-treated cultures (Figure 4A). These transcription factors are unique to the neuronal lineage, control the expression of several downstream genes, and dictate the dopaminergic VM neurons’ morphology, function, and identity [23]. Given that *Lmx1a* and *Lmx1b* have been strongly linked to neural development, it is crucial to understand their precise role in preserving dopaminergic VM neurons [25]. Numerous studies have shown that the transcription factors *Engrailed Homeobox 1* (*En1), Nuclear receptor-related 1 (Nurr1), Pitx3,* and *Lmx1a* play a major role in the early stages of dopaminergic VM neuron formation and are responsible for maintaining the phenotype of adult neurons [26]. Similarly, previous studies have described that *Lmx1a* triggers *Th* activation and enables normal function as mature neurons [27]. In the QEPF-treated cultures, a significant difference was observed in the downregulation of the expression of *Lmx1a* (Figure 4A), *Pitx3* (Figure 4D), *Th* (Figure 4E), *Cell adhesion molecule L1* (*Chl1)* (Figure 4F), *Dopamine transporter* (*Dat)* (Figure 4G) and *Dopamine receptor D1* (*Drd1)* (Figure 4H). In contrast, *Nurr1* (Figure 4B) and *Brain derived neurotrophic factor* (*Bdnf)* (Figure 4I) were significantly upregulated by treatment with QEPF. The expression of *En1* did not change significantly (Figure 4C). Interestingly, the expression of *Pitx3* was affected by exposure to QEPF, which could ultimately alter the early maturation potential of dopaminergic VM neurons. Recent studies have revealed that many genes are critical for the normal development of dopaminergic VM, including *Chl1* (close homolog to *L1*) [27,28]. In this investigation, it was clear that *Chl1* expression in QEPF-treated cultures differed considerably from that in the control cultures (Figure 4F). The proper development of dopaminergic VM neurons may be adversely affected by changes in the expression of the critical genes indicated above.

## 3. Discussion

In the early developmental stages of the brain, the complex signal interplay between the processes of differentiation (specialization) and proliferation (multiplication) tightly governs the size of each developing neuronal cell in the CNS. The ultimate role of VM neurons is determined by the complex signal interplay between the actions of the intrinsic and extrinsic factors. Neurons can respond to an unlimited number of signals, establishing their neuronal connectivity to regulate and operate efficiently within the ongoing cell cycle. Therefore, neurons enter the resting phase of the cell cycle (Go) and become fixed to their ultimate differentiated state and role. These complex cellular events have been studied in both the late and early developmental stages of VM neurons. The dopaminergic VM neuronal population is crucial for proper brain functioning and the development of three vital aspects: behavior, cognition, and motor activity [29]. The present study demonstrated that QEPF at a therapeutic dose of 10 µM had no direct influence on neuronal survival or ATP levels in VM neurons. This study also showed that exposure to QEPF interferes with morphogenesis and structure building in dopaminergic VM neurons during the early stages of neuronal development. This study can influence clinical decision-making regarding the therapeutic use of QEPF in pregnancy. The transcription factors encoded by the genes *Nurr1*, *Drd1*, *Th*, *Bdnf*, *Pitx3*, *Dat*, *Chl1*, and *Lmx1a*, are essential for maintaining regional identity in the midbrain and QEPF significantly altering the expression of each of these genes by either up- or down-regulating their expression. These preliminary findings suggest that future investigations are warranted to confirm the safety of QEPF in pregnancy.

Quetiapine is extensively prescribed for treating and managing schizophrenia, bipolar disorder (BD) (acute episodes and manic states), and depression associated with BD. The indications of quetiapine may also extend to the management and treatment of Alzheimer’s disease, panic attacks, and attention-deficit/hyperactivity disorder (ADHD) [3,30,31,32]. This drug has an exceptional receptor-binding profile and regulates many genes implicated in controlling neuronal cell fate [33]. The effects of QEPF are the opposite of those of the adrenergic, histaminergic, dopaminergic, and serotonergic receptors. Three serotonergic receptors are firmly bound by quetiapine, although dopamine (D1 and D2) and adrenergic receptors only make slight contact [7,34]. Serotonin influences the development of dopamine neurons, and this might be a mechanism to explain the impact of QEPF [35]. Likewise, a more comprehensive analysis of QEPF effects on gene transcription in adult cortical neurons has also been reported [36]. The probability of prenatal quetiapine exposure causing histopathological abnormalities in the embryonic brain remains unknown. An extensive review of the literature on this subject indicates that data on pregnant women’s reactions to QEPF and the development of neurotoxic symptoms in developing fetuses are currently the least well-documented [3]. The one exception was a single study that found the long-term effects of QEPF on neurobehavioral mechanism changes were essentially negligible in young adult mice and their progeny [37]. Therefore, further research is required before QEPF may be regularly prescribed for use during pregnancy to better evaluate safety in pregnant women and its effects on the health of the fetus. This study examined how prenatal exposure to QEPF affects the development and differentiation of dopaminergic VM neurons, which are crucial for controlling emotions, reward systems, drug addiction, voluntary movements, and cognition [38]. QEPF therapy did not affect the viability of VM neurons. Additionally, we observed that a dose of 10 µM QEPF did not disrupt the metabolic activity of VM neurons. This suggests that the medication might not be cytotoxic at a therapeutic dosage (10 µM).

A previous study indicated that antipsychotic drug treatment during pregnancy remains controversial, mainly due to the lack of outcome-based data that would permit a comprehensive risk-benefit assessment [4]. As a result, current treatment guidelines are limited in their applicability to physicians selecting a starting therapy for pregnant women with schizophrenia and physicians’ ability to assess and counsel on accidental exposure to such drugs during pregnancy [4].

We also examined how QEPF affected the structure of dopaminergic VM neurons and discovered that, while the numbers and branching of the neurites were not considerably changed, the lengths of the total and dominant neurites drastically increased following QEPF treatment. In contrast, a different outcome was observed in non-dopaminergic VM neurons, where QEPF did not significantly change the length, branching, or quantity of neurites. Recent transcriptional studies have shown that several genes and factors unique to a particular lineage, including *Pitx3*, *En1*, *Nurr1*, *Lmx1b*, *Th*, and *Lmx1a*, are involved in developing and preserving the functional archetype of dopaminergic VM neurons. [27,39,40]. According to a previous study, *Lmx1a* remains present in mitotic residual precursors and actively specialized neurons in postnatal life, with actual functional value [23]. The gene *Lmx1a* signals and activates *Nurr1* [25], which in turn signals and activates the *Th* gene, further promoting dopaminergic VM neuron expansion [27,41]. Building on this foundation, we investigated how QEPF affected these genes to determine whether it may modify their expression and alter how dopaminergic VM neurons differentiate. The gene expression levels of *Drd1*, *Chl1, Th*, *Pitx3*, *Dat*, and *Lmx1a* were dramatically reduced by QEPF treatment, whereas *En1* expression level was not significantly different. These findings suggest that QEPF decreases the expression of the *Lmx1a*/*Pitx3*/*Th*/*Dat* pathway involved in neuronal expansion. In a previous study, QEPF was shown to possess a moderate affinity for serotonin receptor subtype 5HT2A and alpha-1 adrenergic receptor (α1), amongst other receptors. Furthermore, QEPF has a slight affinity for 5HT1A and dopamine D2 receptors and a lower affinity for alpha-2 adrenergic receptors (α2), 5HT2C, and dopamine D1 receptors [42]. The downregulation of *Th* and *Dat*, shown in this study, could be due to the downregulation of *Lmx1a*, which was shown previously to regulate the differentiation of VM dopaminergic neurons and dopamine transporter [43,44]. Moreover, the downregulation of *Drd1* induced by QEPF might lead to functional disruption and abnormal innervations of VM dopaminergic projections.

Likewise, brain-derived neurotrophic factor (BDNF) is an important growth factor for VM neurons, playing a critical role in remyelination, reversal of neuronal damage, and survival [45]. This study observed that the expression levels of *Bdnf* and *Nurr1* were upregulated significantly upon exposure to QEPF compared to that of the control culture. These findings are similar to a previous study that highlighted the probable mechanism for the efficacy of QEPF in animal models of demyelination through regulating the expression of *Bdnf* and other neurotrophic factors. [46]. Another study concluded that quetiapine regulates the expression of several neurotrophic factors involved in neurogenesis. For instance, it was observed in two studies that QEPF significantly increases BDNF expression levels in the dentate gyri of normal rats and stops decreased BDNF expression in the hippocampus and neocortex of rats, which is provoked by stress-induced immobilization [47,48]. It has also been reported that QEPF treatment inhibits the decrease in BDNF and basic fibroblast growth factor FGF transcription induced by the investigational N-methyl-D-aspartate (NMDA) antagonist MK-801 [49]. Additionally, QEPF prevents the decreased expression of synaptic proteins and BDNF in rat hippocampal neuron cultures under toxic stress conditions induced by a lack of B27 [50].

Overall, the RT-PCR data presented indicates that genes essential for defining the identity of dopamine neurons (*Lmx1a*, *Th*, *Drd1*, *Pitx3*, *DAT*, *Chl1*) are downregulated by QEPF, but *Nurr1* and *Bdnf* (a general driver of neurogenesis) is upregulated. Thus, QEPF may increase neurite outgrowth (likely via *Bdnf*) but impair the differentiation of these cells into functional dopamine-producing neurons. In addition to *Bdnf*, the upregulation of *Nurr1* reported here could also contribute to the elongation of neurites induced by QEPF. Previous studies showed that *Nurr1* is involved in axon genesis in VM dopaminergic neurons [51,52,53]. Moreover, it was demonstrated that *Nurr1* regulates the expression of *Bdnf* in VM dopaminergic neurons [54].

The current study suggests that the upregulation of Nurr1/Bdnf causes an increase in the neurite lengths of VM dopaminergic neurons in response to exposure to QEPF. Ultimately, the findings of this study reinforce and largely corroborate the results of previous studies.

## 4. Materials and Methods

### 4.1. Isolation of Primary Mouse Embryonic VM Dopaminergic Neurons

**Figure 5:**
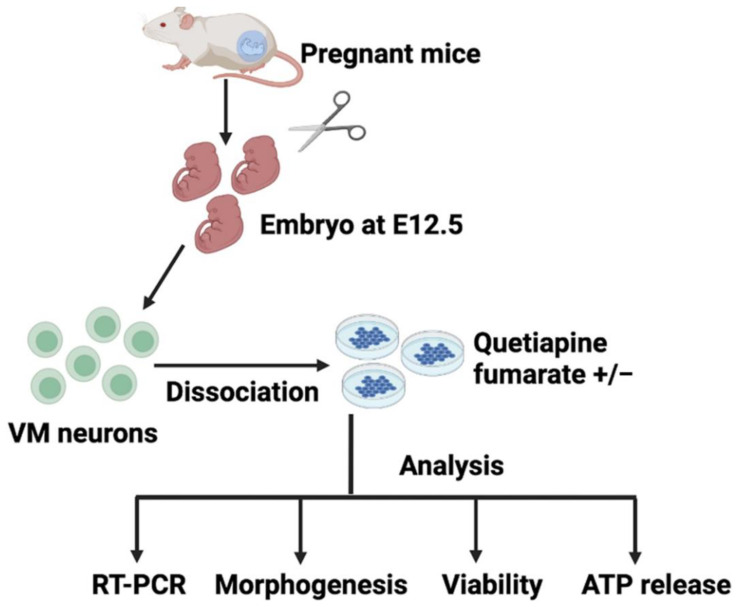
Flowchart depicting the experimental design of this study. VM (Ventral midbrain), RT-PCR (Real-Time PCR), ATP (Adenosine triphosphate).

The Ethics Committee approved the study of King Abdulaziz University (7-CEGMR-Bioeth). All experiments have been conducted in accordance with the international standards of animal use for experimentation and research. Figure 5 shows the detailed experimental design.

Time-mated albino mice were used to collect embryos at the animal house facility of King Fahad Medical Research Centre in Saudi Arabia. The animals were bred overnight and observed the next morning. Embryonic day (E) 0.5 was recorded upon observation of a vaginal plug. In chilled L15 media (ThermoFisher, Waltham, MA, USA), the VM of mouse embryos at E12.5 was excised (*n* = 169 mouse embryos). The border between the telencephalon, mesencephalon, and isthmic organizer was cut to separate the midbrain and most of the cortical tissues. Tissue from the third VM was removed to increase the number of dopaminergic cells in the culture. Hank’s Balanced Salt Solution (HBSS; ThermoFisher Scientific), which is Ca/Mg-free, was diluted with 0.1% DNase (Stem Cell Technologies, Cambridge, MA, USA) and applied for 15 min at 37 °C to separate VMs. The tissues were thoroughly washed three more times in HBSS medium before being re-incubated with N2 medium containing 1 mg/mL bovine serum albumin and a mixture of F12 medium, and Minimum Essential medium with 1 mM glutamine, 1% penicillin/streptomycin, 15 mM HEPES, 6 mg/mL glucose, and 1% N2 supplement (all N2 media components from ThermoFisher Scientific). The in vitro growth of primary neurons was allowed for three days before the experiment, depending on the experiment length (shown in the following text).

### 4.2. Three-Dimensional Neuronal Cell Culture and QEPF Treatment

To imitate normal in vivo development, we selected a three-dimensional (3D) in vitro cell culture system for our experiments. The 3D cultures created in our study were used to test morphogenesis, viability, and adenosine triphosphate (ATP) release in cells and to subsequently perform quantitative PCR. E12.5 primary mouse embryonic VM neurons were plated in 96-well cell culture plates with 6 × 10^4^ cells per well for 3D cultures. We used a rationally designed nonaromatic tetra-peptide amphiphile, Ac-Ac-Ile-Ile-Cha-Lys-NH2 (IIZK), to create 3D cultures according to a previous report. [55]. Dulbecco’s phosphate-buffered saline (DPBS) was used to prepare 1 mg/mL of IIZK- based hydrogels. Previously, this peptide was found to form a stable hydrogel at this concentration in less than seven minutes [16]. Half of the required final volume of nuclease-free sterile water was first used to resuspend the weighed IIZK peptide. Upon addition of DPBS solution, the peptide forms a stable hydrogel that can be used as a scaffold within which the VM neurons can be encapsulated. An appropriate volume of the previously resuspended peptide in water and an equal proportion of 2× DPBS was added to the culture well. A peptide base was applied to each well to prevent the cells from coming into contact with the plastic surface. The plates were incubated for five minutes at 37 °C and 5% CO_2_ to ensure complete gelation. A 3D construct was created on the previously formed cell-free peptide base. The required number of cells was added to 2× DPBS in an equivalent volume along with the peptides, which were then quickly mixed. The plates were incubated again for two to three minutes, and then N2 medium was added to the culture plates. The cells were incubated at 37 °C under 5% CO_2_ for 72 h. QEPF (Sigma, Ronkonkoma, NY, USA) was dissolved in sterile 1× PBS in accordance with the manufacturer’s instructions. After cell seeding, a group of cells was treated with 10 µM QEPF by adding the required volume of the drug into the media within the wells, whereas the control group received an equivalent volume of sterile 1 × PBS. We have determined the dose used here based on the range of plasma and serum concentrations demonstrated in previous studies [42,56,57].

### 4.3. VM Neuronal Viability and ATP Release Assessment

It is crucial to assess how a drug affects the metabolic activity and viability of target cells. After three days of culture, we exposed VM neurons to QEPF to measure their survival and ATP release. We determined the viability of VM neurons in untreated and QEPF-treated cells using alamarBlue™ Cell Viability Reagent (ThermoFisher Scientific, Waltham, MA, USA) as per the manufacturer’s instructions. A PHERAstar FS plate reader (BMG LabTech, Ortenberg, Germany) was used to measure fluorescence after the well plates were prepared. ATP release was measured as a marker of the metabolic activity of cells using the CellTiter-Glo^®^ 3D cell viability assay (Promega, Madison, WI, USA). The 3D construct comprised of the cells and hydrogel was thoroughly mixed by pipetting up and down ten times after CellTiter-Glo^®^ Reagent was introduced in an amount similar to the cell culture medium. A PHERAstar FS plate reader (BMG LabTech, Ortenberg, Germany) was used to scan the plates after 25 min of incubation at room temperature to detect the existence of a strong signal. Three wells from each experiment (seven biological replicates for viability and three for ATP release) were analyzed.

### 4.4. Immunocytochemistry

Mouse embryonic VM neurons were maintained after three days in culture using 4% paraformaldehyde (Santa Cruz Biotechnology, Santa Cruz, CA, USA) and stored at 4 °C in 1 × PBS until staining was completed. Primary antibodies were used to target the enzymes tyrosine hydroxylase (TH) (Cambridge, UK) (ab112) and mouse neuron-specific class III beta-tubulin (TUJ1) (G7121; Promega, Madison, WI, USA). Using the following dilutions, primary antibodies were treated with fixed antibodies—TUJ1 (1:1500) and TH (1:500) in blocking buffer (5% goat serum, 0.3% Triton-X, and 0.2% sodium azide)—overnight at room temperature. The cells were treated with a blocking solution for 1 h at room temperature after removing the primary antibodies. Anti-mouse Alexa 488 and goat anti-rabbit IgG H&L (Alexa Fluor^®^ 555) were added immediately. The secondary antibodies were incubated for 2 h at room temperature after dilution in blocking buffer (1:200). The wells were then cleaned and maintained in 1× PBS, and the cells were treated for five minutes with 4′,6-diamidino-2-phenylindole (DAPI) (D1306; ThermoFisher Scientific, Waltham, MA, USA) and diluted in 1× PBS. Images were obtained using a DMi8 inverted fluorescence microscope (Leica, Wetzlar, Germany).

### 4.5. Morphogenetic Analysis

Administering drugs to neurons while they are still developing may alter their morphogenesis, altering how they connect to their target endogenous ligands in the brain. The effects of QEPF on the growth of VM neurons were assessed in labeled cultures. Analyses of neurite quantity, total neurite length, dominant neurite length, and branch count were performed [28] using the LAS X software (Leica, Wetzlar, Germany). Ten neurons were analyzed in each well (a total of three wells per biological replicate). To avoid research bias, overlapping neurites and those shorter than 20 µm were excluded from the study. Data from the QEPF-treated cultures were normalized to those from the control group. The outcomes were then displayed as a percentage deviation from the control, which was interpreted as a deviation of 100%. The experiment (biological replicates) was repeated four times for dopaminergic VM neurons and six times for non-dopaminergic VM neurons.

### 4.6. Quantitative PCR

The primary developmental process that produces functional adult neurons is neuronal differentiation. It is crucial to assess how a drug affects the ability of target cells to differentiate. The expression of essential genes crucial to the development of this neuronal cell population was examined using an evaluation tool. RNA was extracted after three days of culture using the RNeasy Plus Universal Mini Kit (Cat No. 73404; Qiagen, Hilden, Germany), according to the manufacturer’s instructions. TissueLyser II (Qiagen, Hilden, Germany) was used to homogenize the cells efficiently, according to the RNeasy kit protocol. RNA was isolated from the control and QEPF-treated VM neurons. RNA isolated from tissues other than the brain was used as a negative control. Primer sequences for the selected genes are listed in Table 1.

The Real-Time PCR (RT-PCR) StepOne System and Data Assist software were used to generate raw cycle threshold (CT) data for the housekeeping/reference genes (glyceraldehyde 3-phosphate dehydrogenase (Gapdh) and β-actin) and target genes (tyrosine hydroxylase; Th, nuclear receptor 4A2; Nurr1, LIM homeobox transcription factor 1 alpha; Lmx1a, engrailed homeobox 1; En1, pituitary homeobox 3; Pitx3, dopamine receptor D2; Drd2, and brain-derived neurotrophic factor; Bdnf) in triplicate for both the experimental and negative control groups. The relative gene expression levels were calculated using two reference genes described previously [58]. To assess the expression of target genes under various experimental conditions, fold change (log2FC) was used. log2FC values for each gene in all samples from all groups were compared, and *p*-values were computed to determine whether the genes were substantially expressed. Three wells from each experiment (three biological replicates) were analyzed.

### 4.7. Statistical Analysis

The data are presented as mean ± SEM and compared using Student’s *t*-test by means of GraphPad Prism v 8.1.2 software, with the level of statistical significance set at *p* < 0.05.

## 5. Conclusions

This study aimed to demonstrate the effects of QEPF treatment on dopaminergic VM neurons, which regulate several cognitive and neurobehavioral processes. We examined the mechanisms by which QEPF exposure during pregnancy affects the formation of primary mouse embryonic VM neurons. According to our findings, QEPF exposure interfered with neuron formation and the structural framework of dopaminergic VM neurons during neurodevelopment. In addition, the effect of QEPF on the expression of numerous genes related to the structural development of dopaminergic VM neurons was investigated. The function of adult dopaminergic VM neurons may be altered due to these effects. These findings are encouraging in determining the safety of QEPF during pregnancy and may be crucial in helping clinicians make antipsychotic medication prescribing choices for pregnant individuals. Further studies on cell functioning and in vivo effects of QEPF on dopaminergic VM neurons should be conducted in the future.

## Figures and Tables

**Table 1:** Gene-specific primer pair sequences used in the RT–PCR.

Gene Name	Primer Sequence (5′ to 3′)	
*Gapdh*	Forward-primer: Reverse-primer:	TGA AGG TCG GAG TCA ACG GA CCA ATT GAT GAC AAG CTT CCC G
*β-actin*	Forward-primer: Reverse-primer	GATTACTGCTCTGGCTCCTAGC GACTCATCGTACTCCTGCTTGC
*Th*	Forward-primer: Reverse-primer:	TGA AGG AAC GGA CTG GCT TC GAG TGC ATA GGT GAG GAG GC
*Nurr1*	Forward-primer: Reverse-primer:	GAC CAG GAC CTG CTT TTT GA ACC CCA TTG CAA AAG ATG AG
*Lmx1a*	Forward-primer: Reverse-primer:	GAG ACC ACC TGC TTC TAC CG GCA CGC ATG ACA AAC TCA TT
*En1*	Forward-primer: Reverse-primer:	TCA CAG CAA CCC CTA GTG TG CGC TTG TCT TCC TTC TCG TT
*Pitx3*	Forward-primer: Reverse-primer:	CAT GGA GTT TGG GCT GCT TG CCT TCT CCG AGT CAC TGT GC
*Chl1*	Forward-primer: Reverse-primer:	TGG AAT TGC CAT TAT GTG GA CAC CTG CAC GTA TGA CTG CT
*Dat*	Forward-primer: Reverse-primer:	TTG CAG CTG GCA CAT CTA TC ATG CTG ACC ACG ACC ACA TA
*Drd1*	Forward-primer: Reverse-primer:	CTC AAC AAC ACA GAC CAG AAT GAA CGA GAC GAT GGA GGA
*Bdnf*	Forward-primer: Reverse-primer:	ACT ATG GTT ATT TCA TAC TTC GGT T CCA TTC ACG CTC TCC AGA

## Data Availability

All data supporting the stated results are available in the manuscript.

## References

[1] Toh S., Li Q., Cheetham T.C., Cooper W.O., Davis R.L., Dublin S., Hammad T.A., Li D.-K., Pawloski P.A., Pinheiro S.P. (2013). Prevalence and trends in the use of antipsychotic medications during pregnancy in the US, 2001–2007: A population-based study of 585,615 deliveries. Arch. Women’s Ment. Health.

[2] Leucht S., Corves C., Arbter D., Engel R.R., Li C., Davis J.M. (2009). Second-generation versus first-generation antipsychotic drugs for schizophrenia: A meta-analysis. Lancet.

[3] Singh K., Tripathi N. (2015). Prenatal exposure to a novel antipsychotic quetiapine: Impact on neuro-architecture, apoptotic neurodegeneration in fetal hippocampus and cognitive impairment in young rats. Int. J. Dev. Neurosci..

[4] Abel K.M. (2013). Fetal antipsychotic exposure in a changing landscape: Seeing the future. Br. J. Psychiatry.

[5] Ennis Z.N., Damkier P. (2015). Pregnancy exposure to olanzapine, quetiapine, risperidone, aripiprazole and risk of congenital malformations. A systematic review. Basic Clin. Pharmacol. Toxicol..

[6] Peluso M.J., Lewis S.n.W., Barnes T.R., Jones P.B. (2012). Extrapyramidal motor side-effects of first-and second-generation antipsychotic drugs. Br. J. Psychiatry.

[7] Singh K., Singh M. (2002). Effect of prenatal haloperidol exposure on behavioral alterations in rats. Neurotoxicol. Teratol..

[8] Zhang J., Wang L., Pitts D.K. (1996). Prenatal haloperidol reduces the number of active midbrain dopamine neurons in rat offspring. Neurotoxicology Teratol..

[9] Anderson K.N., Ailes E.C., Lind J.N., Broussard C.S., Bitsko R.H., Friedman J.M., Bobo W.V., Reefhuis J., Tinker S.C. (2020). National Birth Defects Prevention Study. Atypical antipsychotic use during pregnancy and birth defect risk: National Birth Defects Prevention Study, 1997-2011. Schizophr Res..

[10] Hanley G.E., Mintzes B. (2014). Patterns of psychotropic medicine use in pregnancy in the United States from 2006 to 2011 among women with private insurance. BMC Pregnancy Childbirth.

[11] Kennedy D., Eamus M., Hill M., Oei J.L. (2013). Review of calls to an A ustralian teratogen information service regarding psychotropic medications over a 12-year period. Aust. New Zealand J. Obstet. Gynaecol..

[12] Soeiro-de-Souza M.G., Dias V.V., Missio G., Balanzá-Martinez V., Valiengo L., Carvalho A.F., Moreno R.A. (2015). Role of quetiapine beyond its clinical efficacy in bipolar disorder: From neuroprotection to the treatment of psychiatric disorders. Exp. Ther. Med..

[13] Larsen E., Damkier P., Pedersen L., Fenger-Gron J., Mikkelsen R., Nielsen R., Linde V., Knudsen H., Skaarup L., Videbech P. (2015). Use of psychotropic drugs during pregnancy and breast-feeding. Acta Psychiatr. Scand..

[14] Hauser C.A., Zhang S. (2010). Designer self-assembling peptide nanofiber biological materials. Chem. Soc. Rev..

[15] Zhao X., Pan F., Xu H., Yaseen M., Shan H., Hauser C.A., Zhang S., Lu J.R. (2010). Molecular self-assembly and applications of designer peptide amphiphiles. Chem. Soc. Rev..

[16] Susapto H.H., Alhattab D., Abdelrahman S., Khan Z., Alshehri S., Kahin K., Ge R., Moretti M., Emwas A.-H., Hauser C.A. (2021). Ultrashort peptide bioinks support automated printing of large-scale constructs assuring long-term survival of printed tissue constructs. Nano Lett..

[17] Money K.M., Stanwood G.D. (2013). Developmental origins of brain disorders: Roles for dopamine. Front. Cell. Neurosci..

[18] Silverstone P.H., Lalies M.D., Hudson A. (2012). Quetiapine and buspirone both elevate cortical levels of noradrenaline and dopamine in vivo, but do not have synergistic effects. Front. Psychiatry.

[19] Pira L., Mongeau R., Pani L. (2004). The atypical antipsychotic quetiapine increases both noradrenaline and dopamine release in the rat prefrontal cortex. Eur. J. Pharmacol..

[20] Burbach J.P.H., Smits S., Smidt M.P. (2003). Transcription factors in the development of midbrain dopamine neurons. Ann. N. Y. Acad. Sci..

[21] Van den Heuvel D.M., Pasterkamp R.J. (2008). Getting connected in the dopamine system. Prog. Neurobiol..

[22] Parish C.L., Thompson L.H. (2014). Modulating Wnt signaling to improve cell replacement therapy for Parkinson’s disease. J. Mol. Cell Biol..

[23] Doucet-Beaupré H., Gilbert C., Profes M.S., Chabrat A., Pacelli C., Giguère N., Rioux V., Charest J., Deng Q., Laguna A. (2016). Lmx1a and Lmx1b regulate mitochondrial functions and survival of adult midbrain dopaminergic neurons. Proc. Natl. Acad. Sci. USA.

[24] Hobert O., Westphal H. (2000). Functions of LIM-homeobox genes. Trends Genet..

[25] Bergman O., Håkansson A., Westberg L., Belin A.C., Sydow O., Olson L., Holmberg B., Fratiglioni L., Bäckman L., Eriksson E. (2009). Do polymorphisms in transcription factors LMX1A and LMX1B influence the risk for Parkinson’s disease?. J. Neural Transm..

[26] Doucet-Beaupré H., Lévesque M. (2013). The role of developmental transcription factors in adult midbrain dopaminergic neurons. Neurosciences.

[27] Hoekstra E.J., von Oerthel L., van der Heide L.P., Kouwenhoven W.M., Veenvliet J.V., Wever I., Jin Y.-R., Yoon J.K., van der Linden A.J., Holstege F.C. (2013). Lmx1a encodes a rostral set of mesodiencephalic dopaminergic neurons marked by the Wnt/B-catenin signaling activator R-spondin 2. PLoS ONE.

[28] Alsanie W., Penna V., Schachner M., Thompson L., Parish C. (2017). Homophilic binding of the neural cell adhesion molecule CHL1 regulates development of ventral midbrain dopaminergic pathways. Sci. Rep..

[29] Althobaiti Y.S., Almutairi F.M., Alshehri F.S., Altowairqi E., Marghalani A.M., Alghorabi A.A., Alsanie W.F., Gaber A., Alsaab H.O., Almalki A.H. (2021). Involvement of the dopaminergic system in the reward-related behavior of pregabalin. Sci. Rep..

[30] Ahearn E.P., Mussey M., Johnson C., Krohn A., Krahn D. (2006). Quetiapine as an adjunctive treatment for post-traumatic stress disorder: An 8-week open-label study. Int. Clin. Psychopharmacol..

[31] Komossa K., Depping A.M., Gaudchau A., Kissling W., Leucht S. (2010). Second-generation antipsychotics for major depressive disorder and dysthymia. Cochrane Database Syst. Rev..

[32] Stathis S., Martin G., McKenna J.G. (2005). A preliminary case series on the use of quetiapine for posttraumatic stress disorder in juveniles within a youth detention center. J. Clin. Psychopharmacol..

[33] Wang H.-n., Liu G.-h., Zhang R.-g., Xue F., Wu D., Chen Y.-c., Peng Y., Peng Z.-w., Tan Q.-r. (2016). Quetiapine ameliorates schizophrenia-like behaviors and protects myelin integrity in cuprizone intoxicated mice: The involvement of notch signaling pathway. Int. J. Neuropsychopharmacol..

[34] Guzman F. Mechanism of Action of Quetiapine. https://psychopharmacologyinstitute.com/publication/mechanism-of-action-of-quetiapine-2109.

[35] Niederkofler V., Asher T.E., Dymecki S.M. (2015). Functional interplay between dopaminergic and serotonergic neuronal systems during development and adulthood. ACS Chem. Neurosci..

[36] Kondo M., Tajinda K., Colantuoni C., Hiyama H., Seshadri S., Huang B., Pou S., Furukori K., Hookway C., Jaaro-Peled H. (2013). Unique pharmacological actions of atypical neuroleptic quetiapine: Possible role in cell cycle/fate control. Transl. Psychiatry.

[37] Rosengarten H., Quartermain D. (2002). Effect of prenatal administration of haloperidol, risperidone, quetiapine and olanzapine on spatial learning and retention in adult rats. Pharmacol. Biochem. Behav..

[38] Wang M., Ling K.-H., Tan J.J., Lu C.-B. (2020). Development and differentiation of midbrain dopaminergic neuron: From bench to bedside. Cells.

[39] Yin M., Liu S., Yin Y., Li S., Li Z., Wu X., Zhang B., Ang S.-L., Ding Y., Zhou J. (2009). Ventral mesencephalon-enriched genes that regulate the development of dopaminergic neurons in vivo. J. Neurosci..

[40] Volpicelli F., De Gregorio R., Pulcrano S., Perrone-Capano C., di Porzio U., Bellenchi G.C. (2012). Direct regulation of Pitx3 expression by Nurr1 in culture and in developing mouse midbrain. PLoS ONE.

[41] Gyllborg D., Ahmed M., Toledo E.M., Theofilopoulos S., Yang S., Arenas E. (2018). The matricellular protein R-Spondin 2 promotes midbrain dopaminergic neurogenesis and differentiation. Stem Cell Rep..

[42] DeVane C.L., Nemeroff C.B. (2001). Clinical pharmacokinetics of quetiapine. Clin. Pharmacokinet..

[43] Chung S., Kim C.H., Kim K.S. (2012). Lmx1a regulates dopamine transporter gene expression during ES cell differentiation and mouse embryonic development. J. Neurochem..

[44] Doucet-Beaupré H., Ang S.-L., Lévesque M. (2015). Cell fate determination, neuronal maintenance and disease state: The emerging role of transcription factors Lmx1a and Lmx1b. FEBS Lett..

[45] Hyman C., Hofer M., Barde Y.-A., Juhasz M., Yancopoulos G.D., Squinto S.P., Lindsay R.M. (1991). BDNF is a neurotrophic factor for dopaminergic neurons of the substantia nigra. Nature.

[46] Lühder F., Gold R., Flügel A., Linker R.A. (2013). Brain-derived neurotrophic factor in neuroimmunology: Lessons learned from multiple sclerosis patients and experimental autoimmune encephalomyelitis models. Arch. Immunol. Et Ther. Exp..

[47] Xu H., Qing H., Lu W., Keegan D., Richardson J.S., Chlan-Fourney J., Li X.-M. (2002). Quetiapine attenuates the immobilization stress-induced decrease of brain-derived neurotrophic factor expression in rat hippocampus. Neurosci. Lett..

[48] Park S.-W., Lee S.-K., Kim J.-M., Yoon J.-S., Kim Y.-H. (2006). Effects of quetiapine on the brain-derived neurotrophic factor expression in the hippocampus and neocortex of rats. Neurosci. Lett..

[49] Fumagalli F., Molteni R., Bedogni F., Gennarelli M., Perez J., Racagni G., Riva M.A. (2004). Quetiapine regulates FGF-2 and BDNF expression in the hippocampus of animals treated with MK-801. Neuroreport.

[50] Park S.W., Lee C.H., Cho H.Y., Seo M.K., Lee J.G., Lee B.J., Seol W., Kee B.S., Kim Y.H. (2013). Effects of antipsychotic drugs on the expression of synaptic proteins and dendritic outgrowth in hippocampal neuronal cultures. Synapse.

[51] Heng X., Jin G., Zhang X., Yang D., Zhu M., Fu S., Li X., Le W. (2012). Nurr1 regulates Top IIβ and functions in axon genesis of mesencephalic dopaminergic neurons. Mol. Neurodegener..

[52] Kadkhodaei B., Alvarsson A., Schintu N., Ramsköld D., Volakakis N., Joodmardi E., Yoshitake T., Kehr J., Decressac M., Björklund A. (2013). Transcription factor Nurr1 maintains fiber integrity and nuclear-encoded mitochondrial gene expression in dopamine neurons. Proc. Natl. Acad. Sci. USA.

[53] Hegarty S.V., Sullivan A.M., O’keeffe G.W. (2013). Midbrain dopaminergic neurons: A review of the molecular circuitry that regulates their development. Dev. Biol..

[54] Abdollahi M., Fahnestock M. (2022). Nurr1 Is Not an Essential Regulator of BDNF in Mouse Cortical Neurons. Int. J. Mol. Sci..

[55] Alsanie W.F., Bahri O.A., Habeeballah H.H., Alhomrani M., Almehmadi M.M., Alsharif K., Felemban E.M., Althobaiti Y.S., Almalki A.H., Alsaab H.O. (2020). Generating homogenous cortical preplate and deep-layer neurons using a combination of 2D and 3D differentiation cultures. Sci. Rep..

[56] Handley S.A., Bowskill S.V., Patel M.X., Flanagan R.J. (2013). Plasma quetiapine in relation to prescribed dose and other factors: Data from a therapeutic drug monitoring service, 2000–2011. Ther. Adv. Psychopharmacol..

[57] Hasselstrøm J., Linnet K. (2004). Quetiapine serum concentrations in psychiatric patients: The influence of comedication. Ther. Drug Monit..

[58] Vandesompele J., De Preter K., Pattyn F., Poppe B., Van Roy N., De Paepe A., Speleman F. (2002). Accurate normalization of real-time quantitative RT-PCR data by geometric averaging of multiple internal control genes. Genome Biol..

